# Founder reconstruction enables scalable and seamless pangenomic analysis

**DOI:** 10.1093/bioinformatics/btab516

**Published:** 2021-07-14

**Authors:** Tuukka Norri, Bastien Cazaux, Saska Dönges, Daniel Valenzuela, Veli Mäkinen

**Affiliations:** Department of Computer Science, University of Helsinki, Helsinki 00014, Finland; Department of Computer Science, University of Helsinki, Helsinki 00014, Finland; Department of Computer Science, University of Helsinki, Helsinki 00014, Finland; Department of Computer Science, University of Helsinki, Helsinki 00014, Finland; Department of Computer Science, University of Helsinki, Helsinki 00014, Finland

## Abstract

**Motivation:**

Variant calling workflows that utilize a single reference sequence are the *de facto* standard elementary genomic analysis routine for resequencing projects. Various ways to enhance the reference with pangenomic information have been proposed, but scalability combined with seamless integration to existing workflows remains a challenge.

**Results:**

We present *PanVC with founder sequences*, a scalable and accurate variant calling workflow based on a multiple alignment of reference sequences. Scalability is achieved by removing duplicate parts up to a limit into a founder multiple alignment, that is then indexed using a hybrid scheme that exploits general purpose read aligners. Our implemented workflow uses GATK or BCFtools for variant calling, but the various steps of our workflow (e.g. vcf2multialign tool, founder reconstruction) can be of independent interest as a basis for creating novel pangenome analysis workflows beyond variant calling.

**Availability and implementation:**

Our open access tools and instructions how to reproduce our experiments are available at the following address: https://github.com/algbio/panvc-founders.

**Supplementary information:**

Supplementary data are available at *Bioinformatics* online.

## 1 Introduction

The established method for variant calling is to align short reads to some reference genome and then determine the genomic loci for which enough reads support variation with respect to the reference genome. This standard approach suffers from the bias toward the variations chosen for the reference genome (Popejoy and Fullerton, 2016), raising the question of the redesign of the reference (Ballouz *et al.*, 2019). However, as no single reference genome sufficiently represents the genetic variation of individuals of a given species, different alternatives to expand the reference genome have been proposed.

The most straightforward method is to modify the reference genome or to encode variants into it in order to improve read alignment accuracy (Huang *et al.*, 2013; Maciuca *et al.*, 2016; Schröder *et al.*, 2015).

A natural means to express the variants is to represent them with the reference genome using a graph (Computational Pan-Genomics Consortium *et al.*, 2016; Eggertsson *et al.*, 2017; Garrison *et al.*, 2018; Kim *et al.*, 2019; Paten *et al.*, 2017; Pritt *et al.*, 2018; Schneeberger *et al.*, 2009; Sirén *et al.*, 2014, 2020). The nodes of the graph correspond to a subset of the loci and may be numbered with e.g. the reference co-ordinates. Each edge is labeled with the nucleotide subsequence that occurs between the connected loci in e.g. a number of individuals.

Another means is to use a cohort of reference sequences that would represent the variation within a species sufficiently well instead of using only one reference sequence (Danek *et al.*, 2014; Ferrada *et al.*, 2014; Mäkinen *et al.*, 2010; Paten *et al.*, 2017; Wandelt *et al.*, 2013). An advantage of this method is the ability to use existing tools as part of the variant calling workflow as each of the reference sequences may be used as an input to such tools.

In this article, we offer an alternative based on using both multiple reference sequences, as well as generating a modified reference sequence. Our approach is based on combining two novel techniques that together provide scalability and seamless integration to pangenomic resequencing workflows. Namely, we enhance our previous multiple alignment-based pangenomic analysis workflow that already has been shown to work well on difficult genome regions (Valenzuela *et al.*, 2018) with a scalable founder reconstruction algorithm that replaces the huge multiple alignment with a much smaller one made of *founder sequences* (Ukkonen, 2002). We demonstrate that the multiple alignment of founder sequences retains sufficient continuity of the original predicted haplotype sequences so that the workflow is able to produce a very accurate prediction of the individual genome under scrutiny. The accuracy is similar to the graph-based approaches and the approach is also competitive with respect to resource usage.

## 2 Algorithm

**Fig. 1.**
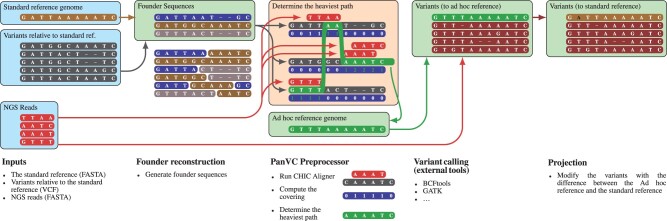
PanVC workflow (Valenzuela *et al.*, 2018) enhanced with founders

Our workflow on a high level consists of the four following steps. Founder sequences are generated from the variants of a group of donors (*Founder Reconstruction*). This saves time and space in comparison to using the predicted haplotype sequences of the donors as is. The reads from a given sample are then aligned to them and, based on those alignments, a single *ad hoc reference sequence* is generated (*PanVC Preprocessor*). A conventional variant calling workflow is then run with the ad hoc reference and the same reads as its inputs (*Variant Calling*). Finally, a co-ordinate transform is applied to the called variants to project them to the original reference co-ordinates, and the variants that are part of the ad hoc reference are reported as well (*Projection*). The workflow is illustrated in Figure 1. The steps are described in the following sections.

### 2.1 A practical algorithm for generating reference sequences from known variation

Reflecting previous work in pangenomics, there are many tools to convert a set of variants stored in *Variant Call Format* (Danecek *et al.*, 2011), VCF, either into a set of sequences or into a graph, but there are no satisfactory solutions to convert them into a multiple alignment. Here we present a method to convert VCF files into a *reference-guided* multiple sequence alignment. That is, rather than studying the NP-hard multiple alignment problem to model homologous regions, we focus on a simpler problem of creating a multiple alignment that is consistent with the given pairwise alignments: Each sample column of VCF file encodes pairwise alignments of its one or more haplotypes against a common reference. While one could obtain a reference-guided multiple sequence alignment using a suitable guide tree in progressive multiple alignment (Durbin *et al.* 1998, Section 6), even the quadratic computation required for pairwise alignments would be prohibitively slow for our purposes. Our approach instead uses the identity regions shared by all pairwise alignments as anchors, and independently re-aligns variants inside regions between consecutive anchors. The re-alignments are optimized for speed, aiming just to obtain a consistent multiple alignment, rather than an optimal one.

We first describe the main ideas behind the algorithm deployed in our implementation and then show how to extend it to generate founder sequences in a practical way.

#### 2.1.1 From variants to a multiple alignment through a directed acyclic graph

Given a set of variant records such as those contained in a VCF file, we observe that records that contain insertions, deletions and polymorphisms may be represented with a directed acyclic graph for each chromosome similarly to previous research on the subject (e.g. Church *et al.* 2015; Dilthey *et al.* 2015). Our idea is to determine the gap positions in the multiple sequence alignment by finding the longest path that ends in a given node by inspecting its in-edges. The output sequences that correspond to shorter paths are then extended with gap characters to match the maximum length.

To create the graph, the first step is to determine every unique starting and ending position of the variants that are located in the chromosome in question. In case the ending position is not included in a variant record, the reference subsequence length added to the starting position is used. For each of these positions, a node labeled with the corresponding genomic co-ordinate is created.

**Fig. 2.**
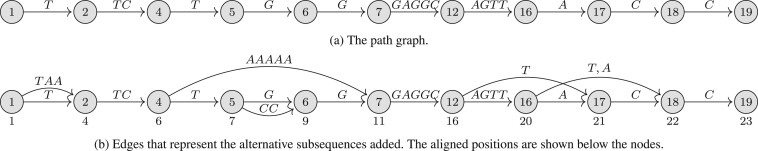
Directed acyclic graph generated from the sample data in Table 1

Suppose *R* is the length of the reference sequence of the chromosome that was chosen. Two additional nodes, 1 and *R *+* *1, are then created to represent the first and last positions if they do not already exist. A path graph is then created by connecting each node except for the last one to the node with the next smallest label by an edge as may be seen in Figure 2a. We call these *reference edges*.

We then consider the alternative subsequences in the variant records. For each such subsequence, an additional edge is drawn from the starting position of the variant to its ending position and labeled accordingly as may be seen in Figure 2b.

Next, an *aligned position* (with respect to the reference-guided multiple alignment) is determined for each node. The aligned position of the first node is 1. For all other nodes, the aligned position is calculated by taking the maximum over the length of the label of each in-edge added to the aligned position of the source node of that edge. These can be computed in linear time in any topological order of the graph.

Certain paths through the graph will now correspond to the samples in the original VCF file. By following such a path, the predicted haplotype sequence of the sample in question may be read by concatenating the edge labels. The reference-guided multiple alignment may also be created by following these paths. After following an edge, the length of its label and the aligned position of the source node are subtracted from that of the target node. The resulting number of gap characters is then appended to the sequence after the edge label. Table 2 shows the result on our running example.

**Table:** Table 1. Sample (artificial) variant data

POS	ID	REF	ALT	s1	s2	s3	s4	s5
1	a	T	TAA	0	0	1	1	0
4	b	TGG	AAAAAA	1	1	0	0	1
5	c	G	CC	0	0	1	1	0
12	d	AGTTA	T	0	0	0	1	1
16	e	AC	T, A	0	1	2	0	0

*Note*: The reference sequence is ‘TTCTGGGAGGCAGTTACC’. The last four columns encode the single chromosome copies for each sample.

**Table 2.** Multiple alignment created from the directed acyclic graph

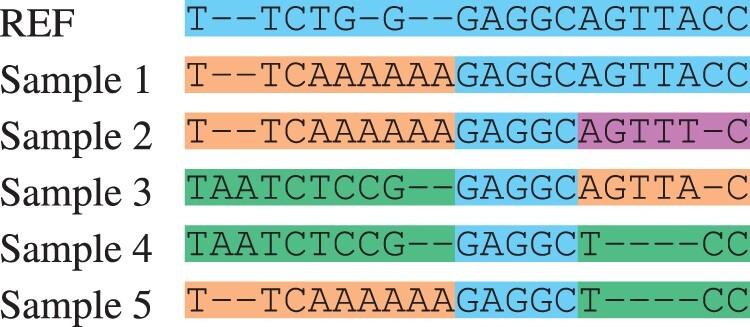

*Note*: Each unique subsequence within a segment has been marked with a different color.

Creating the variant graph may be done in O(v log ⁡o) time where *v* is the number of variant records such that their properties required by the algorithm may be accessed in constant time, and *o* is the maximum number of overlapping variants. O(o log ⁡o) time is needed to sort the variants that have the same starting position. Writing the haplotype sequences to the disk can be done in linear time with respect to the sum of the sequence lengths.

#### 2.1.2 A practical algorithm for generating founder sequences from the directed acyclic graph for read alignment

For our variant graph, all the reference sequences can be read by following a path that goes from the first node to the last node. To align a set of reads on the reference sequences, we must embed the reads into the variant graph. As the number of unique paths of the variant graph, i.e. paths with different sequences of edges, is bounded below by the number of distinct samples in the original variant file, the alignment process may be computationally too expensive.

Instead of creating as many reference sequences as there are samples, one can combine some shared parts of them, a.k.a. *founder segments* (Ukkonen, 2002), as long as the created reference sequences are still useful for read alignment. Norri *et al.* (2019) presented a linear-time algorithm for generating a set of founder sequences from a set of sequences limiting the minimum size of shared founder segments. While the algorithm runs in linear time with respect to the total length of input sequences, the amount of time spent doing I/O operations can be significant. Therefore, in what follows, we develop a practical alternative based on graph bridges. A similar idea based on determining the identity sequences in a multiple sequence alignment was used in the *journaled string tree* (Rahn *et al.*, 2014).

*Optimizing a segmentation in a graph*. A *bridge* in our variant graph is an edge shared by all the paths from the first node to the last. By definition of the graph, this edge is a reference edge. A *bridge node* is a node that has a bridge as an in-edge or out-edge. By splitting the graph by some bridge nodes, we obtain a *segmentation* of the sequences generated from the graph. Then we can consider unique paths only within each segment and create a small number of representative paths by joining the segments. To facilitate the alignment of the reads and reduce the number of reads that map in several segments, only segments with a size (difference between the starting and ending reference positions) of at least *L* are considered where *L* is a user-defined parameter.

Continuing the example in Figure 2b, suppose *L *=* *4. Now suitable cut positions are nodes 7 and 12 as they are connected by a bridge. The lengths of the resulting segments in terms of reference positions are 6, 5 and 7. In general, the label of the connecting edge is not required to be in a separate segment. Since the length of the label in this case is greater than *L*, creating such a segment is possible.

We determine the best choice of cut positions to minimize the number of founder sequences by utilizing a dynamic programming algorithm. To determine the minimum number of paths between the first node and a node *r*, we cut by a bridge node between the first node and *r* and check the minimum number of paths between the first node and the bridge node (dynamic programming condition), as well as between the bridge node and *r*. Formally, we have the following equation:

(1) $$D(r)=\underset{\overset{q-1\geq L}{r-q\geq L}}{\min}\{\max\{D(q),d(q,r)\}\}\;\text{where}\;q,r\in Q$$

where *Q* is the set of bridge nodes and *d* is a function that gives the number of unique paths between two nodes.

To make the algorithm more practical, we utilize branch pruning. If at a given point there are multiple candidate segmentations in which the length of the last segment is at least *L*, the segmentation with the most recently cut final segment is chosen. This does not worsen the segmentation since moving the starting position of the last segment left would not decrease the number of unique paths in it. For listing the cut positions, we maintain a tree of possible cut positions and backtrack from the final leaf node that represents the chosen segmentation. The preprocessing step may be done in *O*(*vwz*) time where *v* is the number of variant records, *w* is the maximum number of active candidate segmentations and *z* is the number of samples.

*Generating founder sequences from a segmentation*. The paths in the segments of the variant graph are joined using a greedy algorithm similar to the one used by Norri *et al.* (2019). Suppose f≥max⁡{d(q,r)} founder sequences are to be generated. Each pair of consecutive segments *s* and *t* is processed from left to right as follows. A bipartite graph G=(S∪T,E) is created with *f* nodes in both *S* and *T*, and set of edges *E* is determined as follows. First each distinct path in *s* and *t* is assigned a node from *S* and *T*, respectively, while the remaining nodes are considered *available*. Then distinct paths in the concatenation of *s* and *t* (that is, the segment that is bounded by the start position of *s* and the end position of *t*) are sorted in descending order by number of occurrences. Each path is then processed by connecting the corresponding nodes in *S* and *T* by an edge. If the corresponding node has already been connected in either *S* or *T* but there is an available node, it will be assigned the path in question and the edge will be drawn. Otherwise, the next path will be considered. When all distinct paths have been processed, the remaining nodes are connected arbitrarily.

To generate the founder sequences, a reference-guided multiple alignment is created like before. To this end, each segment may be processed separately as each pair of consecutive segments is connected by a single edge. The multiple alignment is created for each path represented by the nodes of *S* and *T*, and the sequences are joined as indicated by *E*. If some of the nodes are still available, the corresponding sequences may be filled with N characters. Similarly to the haplotype sequences, writing the founder sequences to the disk can be done in linear time with respect to the sum of the sequence lengths.

Table 3 shows the resulting multiple alignment of the founders in our running example.

**Table 3.** Founder multiple alignment created from the directed acyclic graph

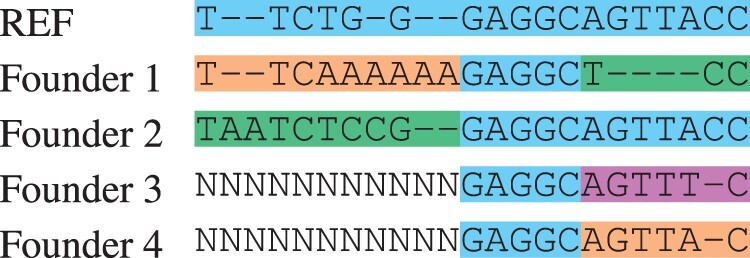

*Note*: There were at most four distinct subsequences within a segment. Since the first segment had only three, one of them was duplicated. As the value of the parameter *L* was 4, each of the subsequences in the second segment begins with the characters GAGG.

### 2.2 Generating an ad hoc reference from alignments to multiple reference sequences

To further compress the generated reference sequences, not all parts of every generated sequence are indexed. Instead, our worklow uses CHIC aligner (Valenzuela and Mäkinen, 2017) to generate a *kernel sequence* (Gagie and Puglisi, 2015) as follows. An *LZ77-compatible parsing* (Valenzuela, 2016) is first generated from the indexed sequences by tokenizing the sequences to two types of subsequences or *phrases*. A *literal phrase* is one that occurs in the set of the reference sequences for the first time and, as such, needs to be represented explicitly. A *copying phrase* corresponds to a subsequence that has occurred earlier and thus may be replaced with a pointer to the previous instance. Consequently, repeating parts of the sequences are replaced with pointers to earlier parts.

Suppose *P* is the length of the aligned reads. The kernel sequence is generated from the LZ77-compatible parsing by processing it from left to right. The literal phrases are copied as they are. When the length of a copying phrase is greater than 2 *P*, only the first and the last *P* characters are copied to the kernel sequence, since any read that would overlap with the middle part of such a phrase may be aligned to the corresponding literal phrase. Our workflow then generates an index from the kernel sequence and aligns the reads to it. We chose to use Bowtie 2 (Langmead and Salzberg, 2012) for this purpose due to its ability to index long reference sequences. Since the kernel sequence only consists of detached phrases with parts of DNA removed from between them, the reads are treated as single-ended at this point.

#### 2.2.1 The ad hoc reference corresponds to the heaviest path through the first-stage alignments

From alignments produced by CHIC, the best alignment is chosen for each read. Suppose there are *m* indexed founder sequences and when these are multiple-aligned, the length of each is *n*. An *m *_**×**_* n* matrix *M* is then created with each cell set initially to zero. If a read aligns best to the *i*th founder sequence at MSA co-ordinates *j* to *k*, then values M[i][j..k] are incremented.

We then process the columns of *M* from left to right and determine the maximum value in each column breaking ties arbitrarily. The character in the same column of the founder sequence that corresponds to that row of *M* is then appended to the ad hoc reference. If the character corresponds to a gap, nothing is added. The process attempts to mimic structural recombinations of the indexed sequences.

### 2.3 Different workflows may be used with the ad hoc reference

After generating the ad hoc reference, different workflows that use a single reference genome could be applied in order to find e.g. structural variants. For our workflow, we chose GATK (Auwera *et al.*, 2013) and followed its best practices, while adding an option to use BCFtools (Danecek *et al.*, 2021). The reads are first aligned to the ad hoc reference using BWA-MEM (Li and Durbin, 2009), treating paired-end reads as such. GATK is then used to remove duplicate reads and finally call variants with HaplotypeCaller.

### 2.4 Called variants are projected to the original reference

Since the founder sequences with which the ad hoc reference was generated may contain other variants than just single nucleotide polymorphisms, the co-ordinates of the ad hoc reference may differ from those of the original reference sequence. A linear-time algorithm is applied to generate a VCF file that contains variants relative to the original reference sequence from the output of the variant caller.

Using matrix *M* the ad hoc reference may be aligned with the multiple sequence alignment of the founder sequences. Suppose that the original reference sequence is also aligned to the other sequences. Then each variant record may be rewritten such that its reference subsequence is set to the corresponding characters in the original reference.

In case the changes made as part of generating the ad hoc reference are supported by the alignments produced by BWA-MEM, the modifications will not be reported in the output of GATK. If this is the case, we determine that the corresponding variant is supported by the aligned reads in every chromosome copy of the sample in question and report it. If a different variant is called by GATK, we inspect the non-zero genotype field values in the variant record, that is, values that indicate that a variant is present in a chromosome copy. The count of such values in the variant record for the sample in question is determined and then subtracted from the ploidy of the organism. Finally, as many genotype field values will be set to non-zero in a new record that corresponds to the difference between the original reference and the ad hoc reference. In addition to that, the variant called by GATK will be reported.

## 3 Methods and discussion

Our overall strategy with the experiments is as follows: The first two experiments with simulated and natural *E.coli* reads aim to show that using founder sequences in place of all haplotypes does not yield decreased accuracy. In addition, the experiments in question aim to reaffirm the benefit of PanVC approach over the use of a single reference on calling variants in difficult to predict regions (Valenzuela *et al.*, 2018). After these experiments, we focus on PanVC with founder sequences only. The second experiment replaces the simulated data used in the first experiment with similar real data, and aims to show that the good behavior of PanVC with founders carries over to a realistic setting. With the lack of truth set, the comparison is more indirect, but we compensate this by using two different quality measures, and compare also to more alternative strategies than the single reference approach. The third experiment continues on real data, but now with a validated truth set (Genome in a Bottle). With this standard dataset, we are able to compare PanVC with founders to the graph-based pangenomic workflows. Despite our best efforts, we were not able to do so for the first two experiments as the tools we tested would not finish either genotyping or building an index with our inputs. Finally, the last experiment is about the scalability, which has been the bottleneck of the original PanVC, and the main motivation for this research.

### 3.1 Experiments with artificial mutations

We tested the accuracy of our variant calling workflow with simulated artificial mutations applied generation by generation to a natural DNA sequence. We believe this method generates variants that would be difficult to predict. Using simulated mutations allows us to generate a sequence to which the called variants could be compared easily; calculating the edit distance of the predicted sequence from the truth would yield a straightforward measure of variant calling accuracy.

To this end, we used the genome of *E.coli* str. K-12 substr. MG1655 as a basis. We started with one simulated bacterium that would have the reference genome. For every generation, each simulated bacterium produced in the previous generation would divide twice and the new bacteria would have some new mutations. Consequently, there would be e.g. 32 bacteria in the fifth generation and 128 bacteria in the seventh generation.

We generated different sets of mutated bacterial genomes by varying the mutation rate. Our mutation model was quite simple: we generated single-nucleotide polymorphisms in random loci. Each locus and each polymorphism was weighed equally.

For the indexing input for our variant calling worklow, we used the generated variants from the fifth, the seventh and the tenth generation of simulated bacteria as well as the reference genome, and for the baseline only the reference genome. In this experiment, we used the haplotype sequences of the generated bacteria as input in addition to founder sequences.

When generating the founder sequences, we tested three values for the parameter *L*: 25, 50 and 100. The number of founder sequences to be generated was determined by taking the maximum number of distinct paths in one segment. The counts are shown in Supplementary Figure S1.

**Fig. 3.**
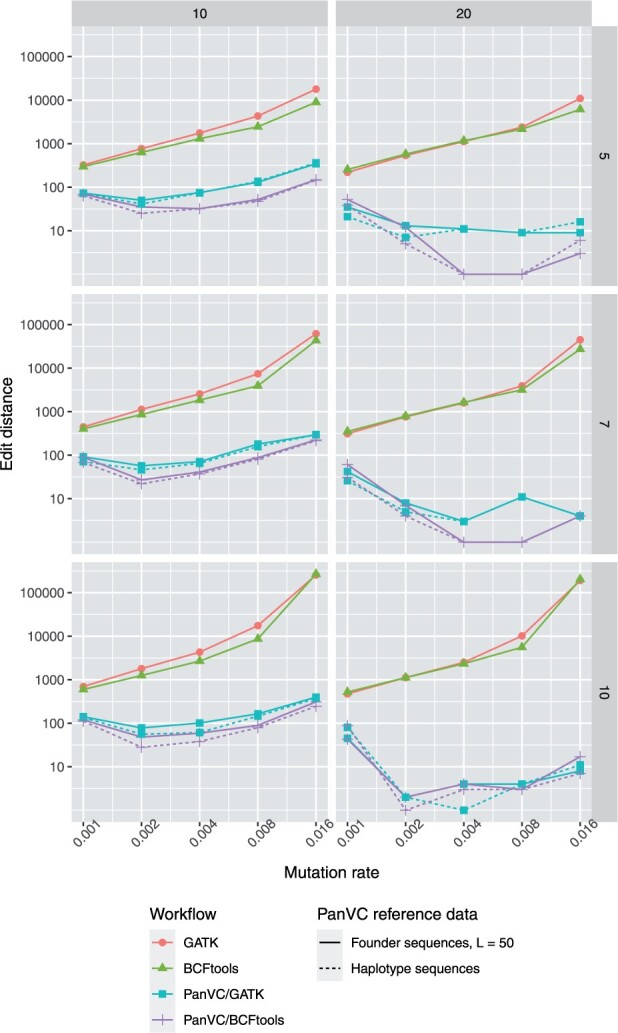
Edit distances for artificial simulated *E.coli* variants in the artificial mutation experiment (smaller is better). Reads have been aligned to an index generated from the same generation samples. The generation number is shown on the right of each row and the read coverage is shown on the top of each column. With PanVC, both founder sequences (solid line) and haplotype sequences (dashed line) were tested

We then ran all six workflows (GATK and BCFtools with the initial reference genome and with the two ad hoc references generated with PanVC using haplotype sequences and with PanVC using founder sequences) with reads generated from the variants of one bacterium from the same generation. We used paired-end reads of 100 bases with 1% error rate and varied coverage. From the called variants, we generated the predicted sequence of the bacterium and calculated the edit distance to the original with Edlib (Šošić and Šikić, 2017). The results for *L *=* *50 compared to the other workflows are shown in Figure 3. Other results are shown in Supplementary Figures S2 and S3.

#### 3.1.1 Variants become easier to predict with multiple reference sequences when mutation rate is moderately increased

In all cases, our predicted sequence was significantly closer to the original than the baseline. E.g. with 20× coverage the predicted sequence generated from variants called by GATK had edit distance of 1116 from the original while our prediction with the same variant caller had edit distance of 1 when using the variants from the tenth generation of simulation with mutation rate of 0.002.

We got consistently better results by using a variant calling workflow based on BCFtools instead of GATK in terms of edit distance to the original sequence. We believe this to have happened due to the random nature of the generated variants.

Using founder sequences instead of all haplotype sequences of the related samples did not affect the results negatively.

#### 3.1.2 Choosing a suitable value for *L*

While increasing the value of *L* reduces the number of segment boundaries in the founder sequences, it also causes the number of generated founder sequences to approach that of the original samples. On the other hand, decreasing the value leads to short segments, which affects the variant calling results negatively. As the segment boundaries occur more frequently, the number of generated founder sequences would have to be increased in order to increase the likelihood of a subsequence matching to a given read occurring in the index.

Based on our results, we determined that choosing *L *=* *50 would produce results similar to generating an index from all of the haplotype sequences of the related samples. Different values of *L* did not affect the results of the second and the third experiment notably.

### 3.2 Experiments with natural *E.coli* samples

In our second experiment, we used 20 samples of real *E.coli* bacteria in order to show that our workflow produces good results with natural data. Since comparing the variant calling results to truth would be difficult, as the DNA sequences of the bacteria in the samples were not known, we decided to inspect the read alignment results. The sequencing data were downloaded from Sequence Read Archive (SRA) maintained by National Center for Biotechnology Information, Bethesda, MD, US. The identifiers of the data are listed in Supplementary Table S2.

To produce an index, we chose 99 reference sequences of different strains of *E.coli* bacteria and generated generate pairwise alignments with each and *E.coli* K-12 substr. MG1655 using Edlib aligner (Šošić and Šikić, 2017). We treated the output as variants with respect to *E.coli* K-12 and generated founder sequences from it, and finally indexed the resulting 20 sequences with PanVC. The strains are listed in Supplementary Table S1.

We compared PanVC with founders to three other workflows. In the first workflow (*Baseline*), we used only a single *E.coli* K-12 substr. MG1655 reference sequence for indexing. Similarly to PanVC’s second stage, BWA-MEM was used to align the reads. For the second alternative workflow (*Consensus*), we chose 19 random *E.coli* reference sequences from the set of 99 references and added that of *E.coli* K-12 to the set. We then generated a consensus sequence with BCFtools (Danecek *et al.*, 2021) from the pairwise alignments from which the founder sequences were generated for indexing. We used a simple dynamic programming algorithm to maximize the allele counts of the alternative subsequences while removing any overlaps. Similarly to the baseline workflow, the reads were aligned with BWA-MEM. For the third alternative workflow *(PanVC (haplotypes)*), we used the same 20 reference sequences as indexing inputs but generated the index with PanVC. The reads were aligned by running the PanVC workflow.

**Fig. 4.**
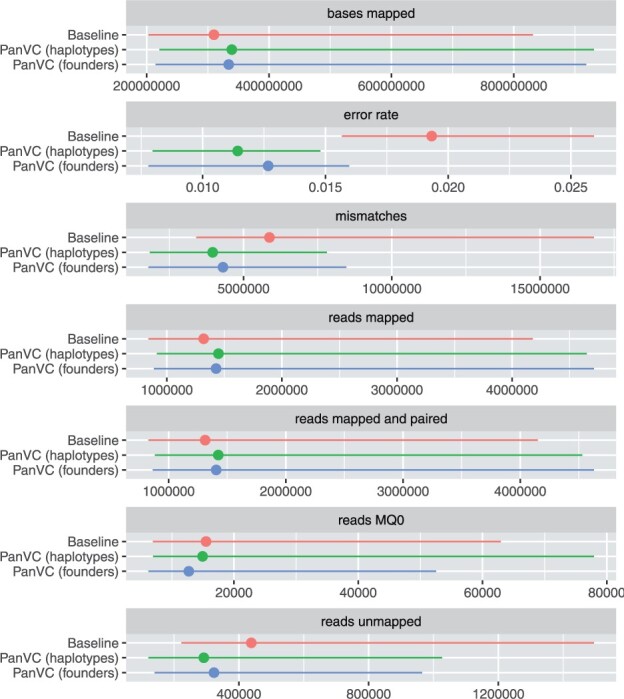
Alignment statistics as reported by Samtools in the first part of the *E.coli* experiment with natural reads. Each line shows the range of values with the median value marked. ‘Reads MQ0’ denotes reads with zero mapping quality. The results for the consensus workflow have been omitted due to the small number of mapped reads

We evaluated the alignment results with Samtools’s stats command (Danecek *et al.*, 2021). A summary of the statistics is shown in Figure 4.

**Fig. 5.**
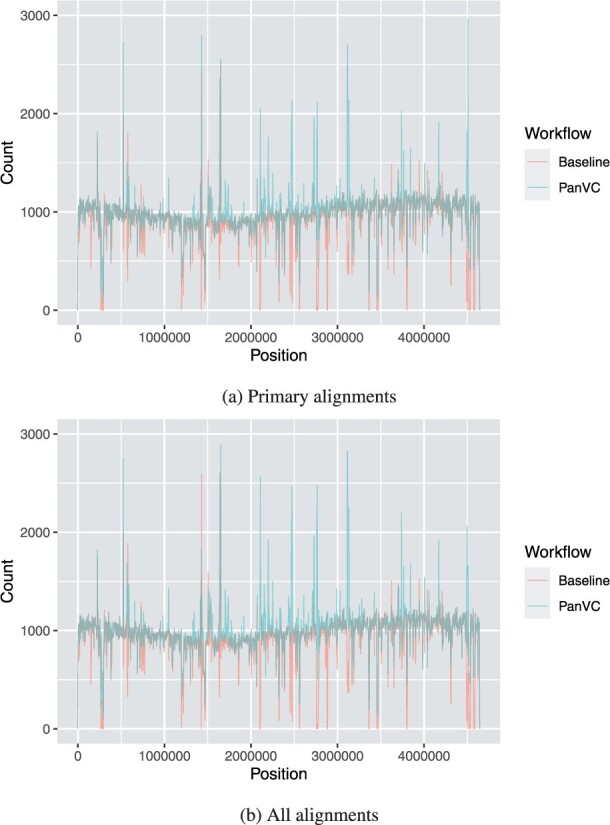
Average number of mapped reads per position (all 20 samples combined) in the first part of the *E.coli* experiment with natural reads considering the starting positions only. Primary alignments (**a**) and all alignments (**b**) for PanVC and baseline workflows are shown. The bucket width is 2500

For PanVC with founders and baseline workflows, we also inspected the mapped segments that passed the filters and were not duplicates. In case of PanVC, we converted the alignment co-ordinates back to those of *E.coli* K-12. Finally, we visualized the mapping coverage by plotting frequency polygons of the average number of segments per position. We considered only the leftmost position of each segment instead of all positions covered. Only primary alignments were included in one plot, and all alignments in another. The results are shown in Figure 5a and b.

**Fig. 6.**
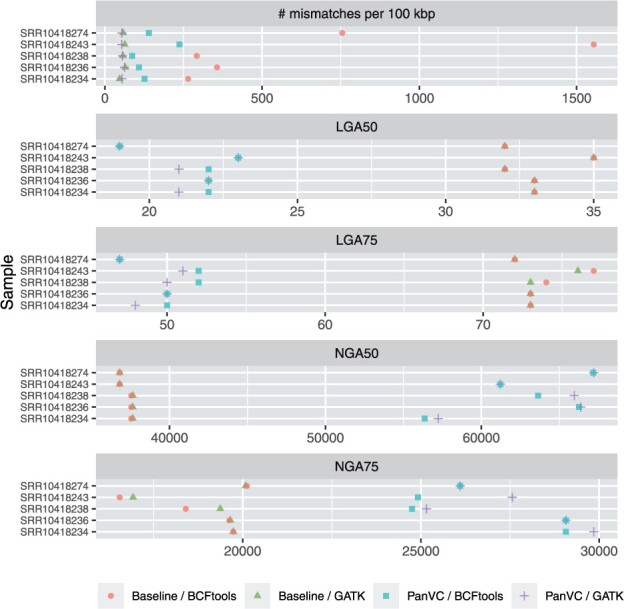
Quality assesment results in the second part of the *E.coli* experiment with natural reads as reported by QUAST

To supplement our analysis, we chose five more samples from SRA for the strains of which *de novo* sequenced contigs were available. We used QUAST (Mikheenko *et al.*, 2018) to evaluate these by using the predicted sequences from PanVC with founders and baseline workflows as references. Our hypothesis is that the *de novo* sequencing quality would be better if the predicted sequence were closer to the truth. We considered three metrics: the length for which the collection of all aligned blocks of that length or longer covers at least a given proportion of the reference genome (‘NGAx’), the minimal number of aligned blocks that cover at a given proportion of the reference genome (‘LGAx’) and the number of mismatches per 100 kbp. The results are shown in Figure 6. The samples used are listed in Supplementary Table S3.

#### 3.2.1 Better mapping results are achieved with PanVC

The statistics collected with Samtools indicate that the alignment results were favorable for PanVC compared to baseline: The number of mapped reads and bases were higher. The number of unmapped reads and reads with zero mapping quality, as well as the number of mismatches were lower.

Differences between PanVC with random haplotypes and PanVC with founder sequences were quite small. Indeed, using a random subset is another way of making PanVC scalable. However, we propose using founder sequences for this purpose as they are a deterministic and systematic means to take into account all haplotypes in the input. We also note that the number of reads with zero mapping quality was smaller when PanVC was used with founder sequences compared to either baseline or PanVC with haplotypes.

While in the alignment results of both PanVC and baseline there are regions to which a smaller number of reads have been mapped, PanVC’s results show smaller differences among regions. When determining the heaviest path, PanVC takes deletions supported by the alignments into account while insertions are ignored. However, based on our results, this has not caused an increase in unmapped regions.

In case of the consensus workflow, very few reads were mapped (some 3000 to 22 000 out of 1 million to 5.7 million depending on the sample). We believe this may have had to do with the large number of variants in the indexing input; there were some 300 000 loci out of 4.6 million that were not a starting position of a variant even after removing the overlapping variants.

Comparison done with QUAST also indicates that the *de novo* sequenced contigs had fewer mismatches with the predicted sequence of PanVC compared to that of baseline when BCFtools was used for variant calling. When GATK was used the differences in the number of mismatches were small. The other metrics showed that the number of aligned blocks needed to cover a proportion of the predicted sequence generated with PanVC was smaller than that generated with baseline. Similarly, the minimum length of the aligned blocks in the set that is needed to cover at least a proportion of the predicted sequence generated with PanVC was greater than that generated with baseline.

### 3.3 Take-one-out experiment with a human chromosome

A natural step was to test our workflow with human data. We simulated a situation where the whole genome of an individual is sequenced but variant analysis is done on one chromosome only. We used the sequencing data for NA12878 from Illumina Platinum Genomes project (Eberle *et al.*, 2017) as input and the variant calls for the individual from the Genome in a Bottle project (Zook *et al.*, 2016) as a benchmark, considering confident regions only. To save computing resources, we took a sample of approximately half of the reads to lower the read coverage to approximately half of the original.

We used the phase 3 variant data relative to the hs37d5 reference from the 1000 Genomes Project (The 1000 Genomes Project Consortium *et al.*, 2015) to create an index for PanVC. We removed NA12878 and their close relatives and generated founder sequences for chromosome 1.

The value for *L* for generating the founder sequences was determined by repeating the experiment while varying the value of the variable. After generating the founder sequences, we built an index with PanVC, executed the workflow and evaluated the results with hap.py (https://github.com/Illumina/hap.py). Based on the results we determined that the known variation is represented sufficiently well with a very small number of founders. We set *L *=* *2 and generated two founder sequences for chromosome 1 in addition to the reference sequence.

For comparison we chose three other workflows: A baseline workflow used hs37d5 as the reference sequence. Second alternative workflow used GraphTyper 2 (Eggertsson *et al.*, 2017) for processing the alignments produced by BWA to call variants. We post-processed the variants as suggested by the authors by marking only the variants with an AAScore value of 0.5 or higher as passing. Third alternative workflow used vg (Garrison *et al.*, 2018) for read alignment and GATK for variant calling. All variant calls were evaluated with hap.py.

**Fig. 7.**
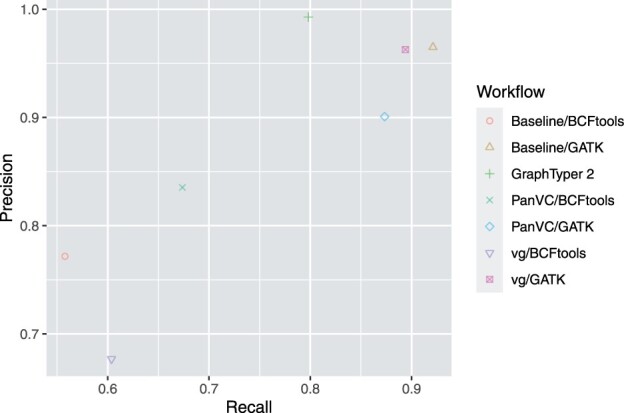
Indel genotyping precision and recall for confident regions as reported by hap.py in the human chromosome 1 experiment. Only variants passing all filters were considered

The results are shown in Table 4 and Figure 7.

**Table 4.** SNP genotyping precision and recall for confident regions as reported by hap.py in the human chromosome 1 experiment

Workflow	Precision	Recall
Baseline/GATK	99.73%	99.71%
Baseline/BCFtools	99.76%	99.30%
PanVC/GATK	99.62%	99.73%
PanVC/BCFtools	99.58%	99.48%
GraphTyper 2	99.86%	95.56%
vg/GATK	99.30%	96.74%
vg/BCFtools	99.58%	95.90%

*Note*: Only variants passing all filters were considered.

#### 3.3.1 Higher precision and recall for genotyping SNPs are retained with PanVC

Our results show small differences in SNP genotyping results among PanVC and baseline workflows (Table 4). Applying PanVC slightly decreased precision with respect to baseline and slightly improved recall. GraphTyper 2 and vg had a somewhat lower recall compared to both PanVC and baseline.

For indels (Fig. 7), our results indicate that PanVC improved both precision and recall with respect to the baseline workflow when BCFtools was used for variant calling. Considering the other workflows, Graphtyper 2’s precision was the highest. When GATK was used for variant calling, PanVC had a better recall than GraphTyper 2 but both its precision and recall were lower than those of vg. Overall best results were attained with the baseline workflow; note that the success of GATK on this dataset is inevitable as it has been an integral part in the generation of the ground-truth used in this experiment (Zook *et al.*, 2016).

### 3.4 Scalability experiment with the whole human genome

To test the scalability of our workflow, we used reads from ERR1025645 sequencing run from Simons Genome Diversity Project (Mallick *et al.*, 2016). We took a random sample of approximately half of the reads to reduce the coverage to approximately 20, ran the PanVC workflow and compared the read mappings to those produced with BWA-MEM. With PanVC, we used the founder sequences generated for each chromosome from 1000 Genomes Project data. We set *L *=* *30 and generated 65 founder sequences for each chromosome. The reference sequence used for this purpose, as well as aligning the reads with BWA-MEM, was hs37d5 excluding the decoy sequences.

The machine used for this experiment was a server equipped with 96 Intel(R) Xeon(R) CPU E7-4830 v3 processors running at 2.10 GHz. We measured the time and memory required and for the latter separated the steps that are specific to our workflow. As the time and memory usage were measured per-process, we used the maximum time when the number of steps done in parallel was smaller than the number of processors, and otherwise calculated an average time spent by one processor.

At the same time, the proportions of mapped reads as well as the edit distances of the reads were determined by inspecting the alignment flags in the resulting BAM files. The results are shown in Table 5 and Figure 9.

**Table 5.** Proportion of mapped reads by workflow in the scalability experiment

Workflow	Mapped reads	Properly paired
Baseline	99.44%	97.42%
PanVC	99.45%	97.36%

#### 3.4.1 Using PanVC adds moderately to the wall clock time required for variant calling

**Fig. 8.**
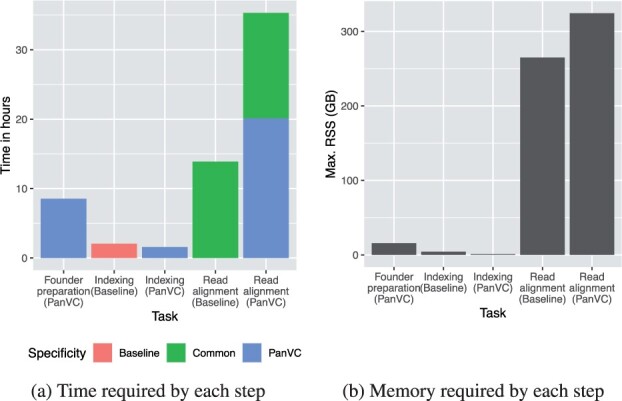
Time in hours and memory in gigabytes required to generate an index and align a sample of reads with 20× coverage in the scalability experiment. The steps as categorized in the figures are indexing and read alignment for baseline, and founder preparation, indexing and read alignment for PanVC. The lower part of the rightmost column labeled *PanVC-specific* in the timing plot indicates the steps that are specific to PanVC while the remaining part shows the steps that are common to PanVC and a single-reference workflow

The timing results are shown in Figure 8a and the peak memory usage is shown in Figure 8b. Using PanVC as part of read alignment increased the total duration by some 20 h. The smaller amount of time required by PanVC for indexing is explained by the fact that the LZ-compatible parsing generated from the reference sequences is quite space-efficient, and indexing the ad hoc reference is done as part of read alignment. The read alignment done by Bowtie 2 took in this case around 13 h. A post-processing step that took 4 h was, on the other hand, implemented in Python and is therefore a candidate for optimization.

#### 3.4.2 *Ad hoc* reference is closer to the ground truth

**Fig. 9.**
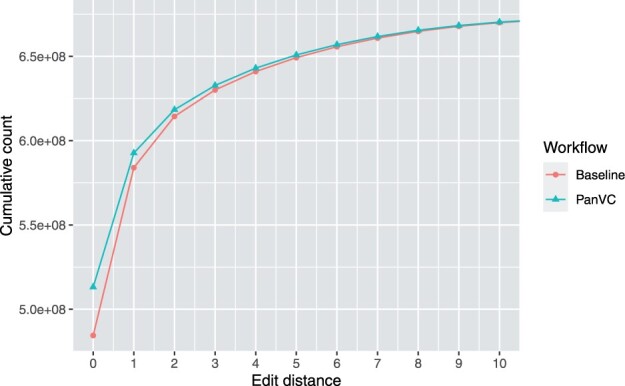
Mapped read count by maximum edit distance and workflow in the scalability experiment. Edit distances from zero to ten are shown. The difference in counts between PanVC and baseline at position 0 is approximately 28.7 million

As seen in Table 5 our workflow maps more reads in total, but with a slight cost in the accuracy of paired alignments. Figure 9 shows that our workflow maps more reads with no errors, indicating that making use of the ad hoc reference results in better alignment locations for the reads.

## 4 Conclusion

In our experiments, we tested our approach of using both multiple reference sequences as well as generating a modified reference sequence. We have shown that the approach yields good results in different types of scenarios. The precision and recall of our predicted variants is good compared to those received from both conventional and graph-based workflows. When tested with a set of reads generated from a sample that contained a high number of mutations, our prediction of the DNA sequence of the sample was very close to the truth. However, our attempts to show similar improvements with real sequencing data of human genome ended up with somewhat equivocal results: On a ground-truth data based on variant calls from a standard reference, best results were attained using no pangenomic information at all. On another real dataset, better read alignments were achieved using PanVC when compared to a standard workflow.

We believe that the workflow achieves the good results by utilizing existing tools that are tried-and-tested for variant calling. Since the tools that we developed can be integrated to other conventional workflows, it is possible to improve their accuracy especially in handling difficult genomic regions.

When generating the founder sequences, we considered allele phasing in that alleles from the same chromosome copy would occur in the same path. A natural direction to which improve our approach would be considering whether some variants occur in the same chromosome copy and placing the variants in question to the same founder sequence. The idea could be extended by generating one ad hoc reference sequence for each homologous chromosome. This would require support from other tools that are part of the workflow, though.

Scalability of the workflow can be further improved by adding support to distributing the workload to a cluster. This has already been accomplished for the original PanVC workflow using Spark (Maarala *et al.*, 2020), and we are working with our collaborators to extend the cluster support to include the new founder reconstruction-related parts.

While scaling the approach with founder sequences, we noticed that in some cases we can also improve the accuracy by using fewer founders. We believe that our algorithm for determining the heaviest path may need to be improved for handling identical regions in multiple reference sequences to solve this issue. Such improvement would make the approach more robust to different parameter choices (e.g. minimum length of founder segment). We briefly tested some alternatives: One option was to choose the highest-scoring nucleotide for each position of the ad hoc reference instead of taking the nucleotide from the founder sequence with the highest score. Another was to use a dynamic programming algorithm to limit switching from one founder sequence to another. However, the current algorithm yielded the best variant calling results so far.

Another important future extension to our workflow is adding support to structural variants. While graph-based pangenome representations may extend more naturally to encode such variants (Eggertsson *et al.*, 2019; Hickey *et al.*, 2020), we believe our multiple alignment-based framework also works by just encoding all structural variants as insertions; this will yield long gap regions in the (founder) multiple alignment, but in the end affects only the internal data structures that are used for projecting the aligned loci, as well as the routines to report the results (from insertions back to the structural variant). Thus, some engineering may be required to adjust and optimize the component of the workflow to a given set of structural variants.

## Supplementary Material

btab516_Supplementary_Data
