# Supplementary material for: Founder reconstruction enables scalable and seamless pangenomic analysis
Source: Bioinformatics. 2021 Jul 14;37(24):4611–9. doi: 10.1093/bioinformatics/btab516 (PMC8665761; doi:10.1093/bioinformatics/btab516)
Supplement: btab516_Supplementary_Data [file btab516_supplementary_data.pdf]

Supplement: Founder Reconstruction Enables Scalable and Seamless Pangenomic Analysis

Tuukka Norri, Bastien Cazaux, Saska Dönges,
Daniel Valenzuela, and Veli Mäkinen

May 2021

1 Additional experimental results

1.1 Experiments with artificial mutations

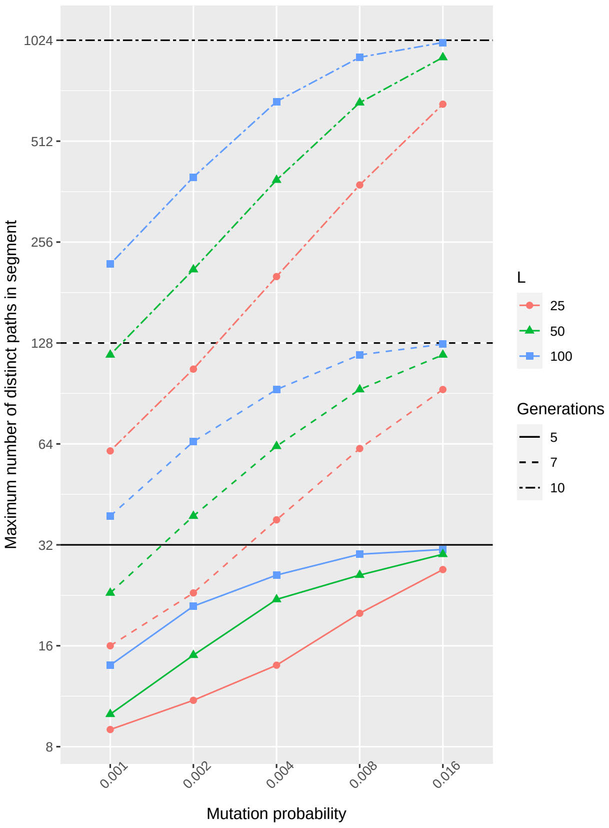

Supplemental Figure 1: Maximum number of distinct paths in one graph segment in index data for the artificial mutation experiment (smaller is better). Index data for all generations is shown. The number of samples in each case in shown with a horizontal line.

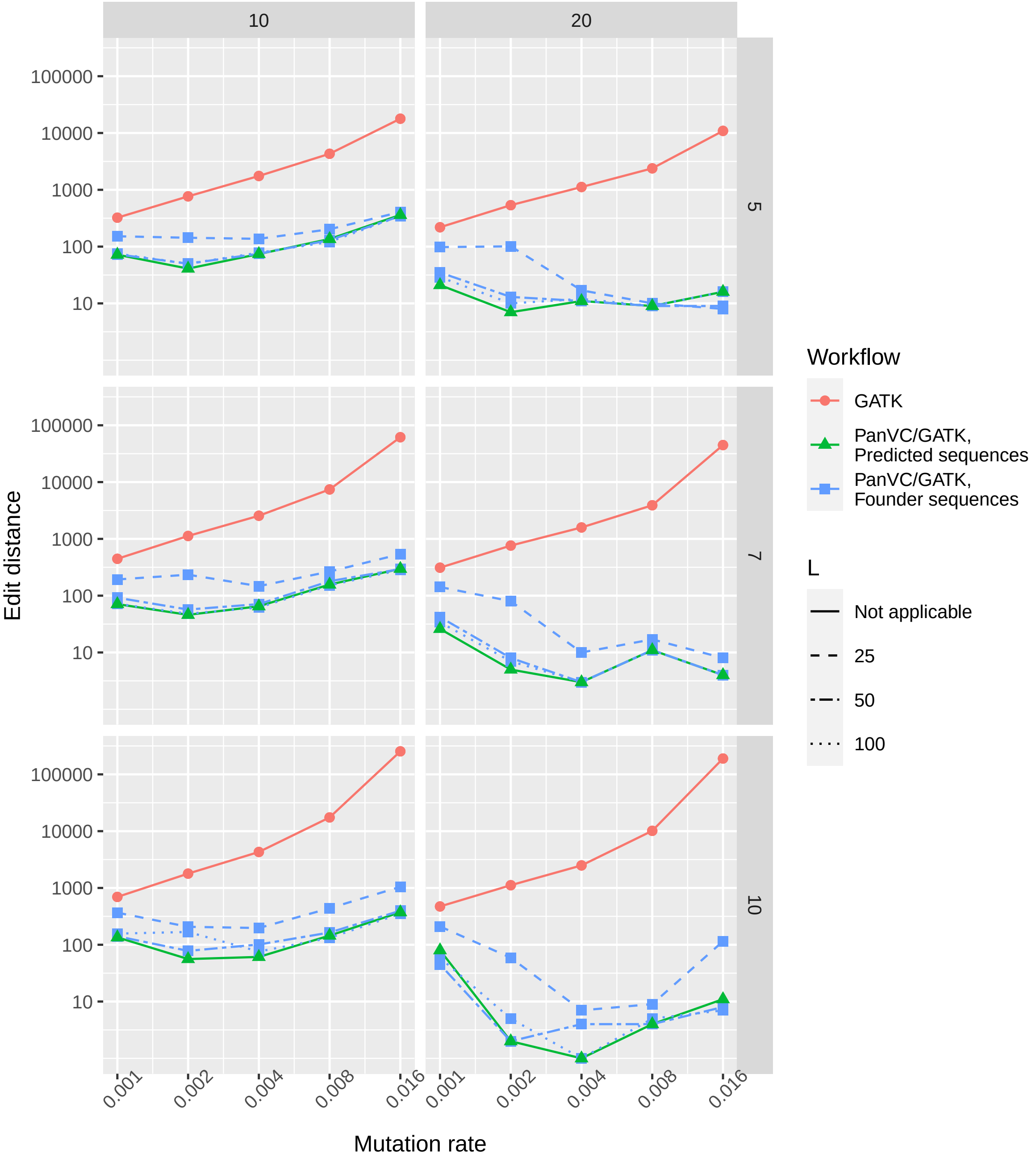

Supplemental Figure 2: Edit distances in the artificial mutation experiment when using GATK for variant calling. Founder sequences were generated with values 25, 50 and 100 for the parameter L. Read coverage is shown on the top of each column and generation number is shown on the right of each row. Results were similar to those with all of the predicted sequences when L was set to either 50 or 100.

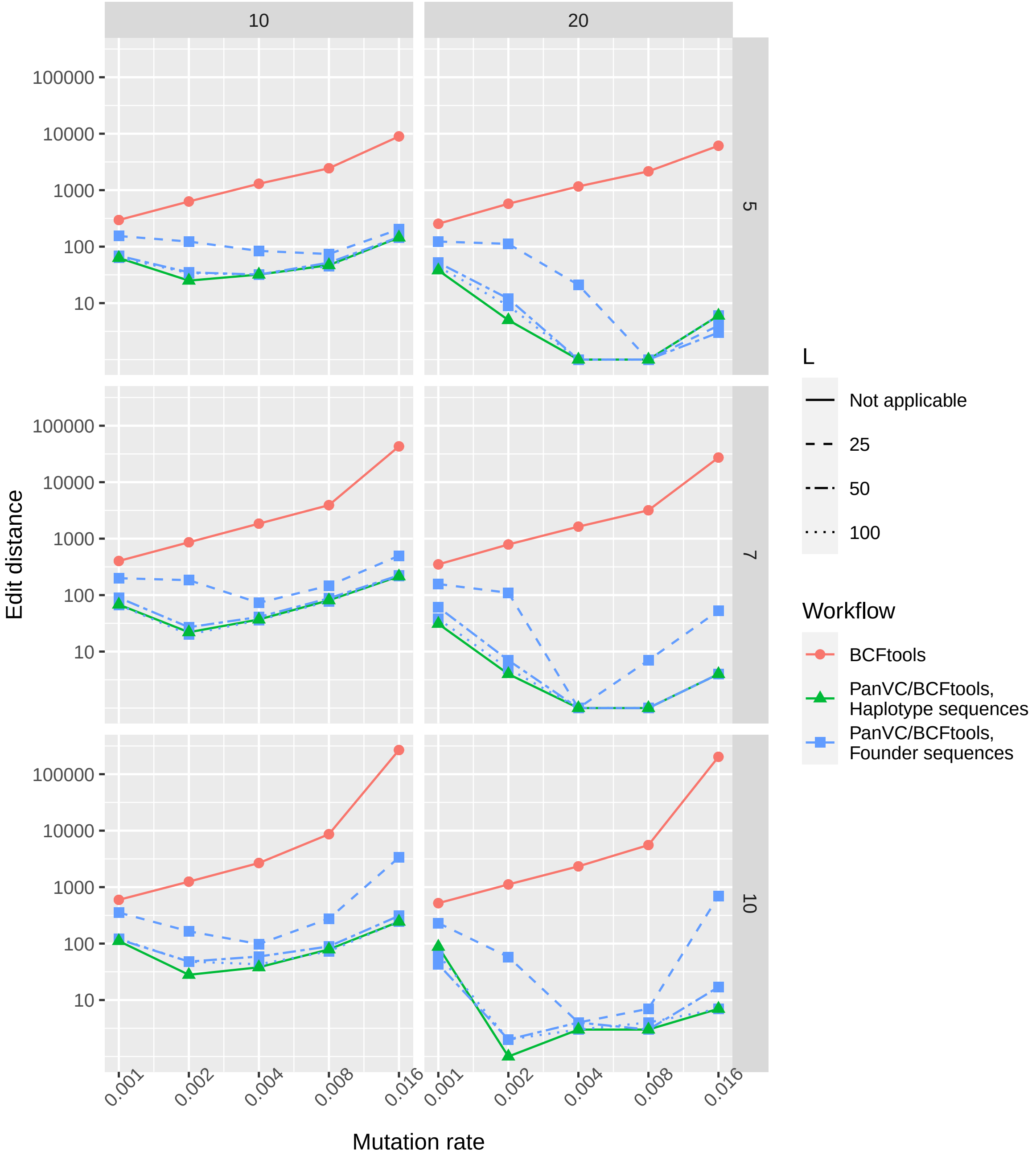

Supplemental Figure 3: Edit distances in the artificial mutation experiment when using BCFtools for variant calling. Founder sequences were generated with values 25, 50 and 100 for the parameter L. Read coverage is shown on the top of each column and generation number is shown on the right of each row. As with GATK, results were similar to those with all of the predicted sequences when L was set to either 50 or 100.

1.2 Experiments with natural E.coli samples

Supplemental Table 1: The strains of the reference sequences used in the experiment with natural E.coli samples. Strains used for the consensus sequence and for PanVC with haplotypes are shown in boldface.

Organism Name	Strain	Sample	Bioproject
**E.coli str. K-12 substr. MG1655**	**K-12 substr. MG1655**	**SAMN02604091**	**PRJNA225**
E.coli O157:H7 str. Sakai	Sakai substr. RIMD 0509952	SAMN01911278	PRJNA226
E.coli DSM 30083 = JCM 1649 = ATCC 11775	ATCC 11775	SAMN10252913	PRJNA472652
E.coli	97-3250	SAMN08638904	PRJNA218110
E.coli O157	644-PT8	SAMN05007045	PRJNA321984
E.coli	2015C-4944	SAMN04482320	PRJNA218110
E.coli O26 str. RM10386	RM10386	SAMN08724660	PRJNA69935
E.coli O157:H7	FRIK944	SAMN04510563	PRJNA322195
E.coli	MBT-5	SAMN15423745	PRJNA643697
**E.coli**	**FORC_028**	**SAMN04026657**	**PRJNA294502**
E.coli O26:H11 str. 11368	11368	SAMD00060955	PRJDA32509
E.coli O26:H11	FWSEC0001	SAMN08768102	PRJNA287560
E.coli O145:NM	FWSEC0002	SAMN08768103	PRJNA287560
E.coli	CFSAN027343	SAMN10604454	PRJNA230969
**E.coli**	**2013C-4538**	**SAMN08579598**	**PRJNA218110**
E.coli	E2865	SAMD00129668	PRJDB7170
E.coli	2014C-3050	SAMN03569438	PRJNA218110
E.coli O26 str. RM8426	RM8426	SAMN08724651	PRJNA69927
**E.coli**	**2012C-4606**	**SAMN04498549**	**PRJNA218110**
E.coli	2013C-4225	SAMN08579596	PRJNA218110
E.coli	2014C-3057	SAMN08579576	PRJNA218110
E.coli O157:H7	FRIK2455	SAMN04531998	PRJNA322195
E.coli O157:H7	FRIK2069	SAMN04191551	PRJNA322195
**E.coli O157:H7**	**FRIK2533**	**SAMN04191553**	**PRJNA322195**
E.coli O157:H7	SS TX 313-1	SAMN05360235	PRJNA528413
E.coli	2013C-3252	SAMN02991246	PRJNA218110
E.coli O103 str. RM8385	RM8385	SAMN08724650	PRJNA69931
E.coli	RM13322	SAMN14449600	PRJNA573729
E.coli	CAU16175	SAMN13705263	PRJNA598281
**E.coli O157:H7**	**BB24-1**	**SAMN05360196**	**PRJNA528413**

E.coli O157:H7	NE 1092-2	SAMN05360231	PRJNA528413
E.coli	143	SAMN08773050	PRJNA445267
**E.coli O157:H7**	**pv15-279**	**SAMD00108492**	**PRJDB6584**
**E.coli**	**2014C-3051**	**SAMN08579564**	**PRJNA218110**
E.coli O157:H7	Show KS 470-1	SAMN05360225	PRJNA528413
E.coli O157:H7	F6294	SAMN11229489	PRJNA528413
E.coli	CFSAN027346	SAMN10604455	PRJNA230969
E.coli O157:H7	TX 265-1	SAMN05360242	PRJNA528413
**E.coli O145:H28 str. RM13514**	**RM13514**	**SAMN02641383**	**PRJNA178648**
**E.coli O145:H28 str. RM12581**	**RM12581**	**SAMN03081527**	**PRJNA178647**
**E.coli**	**2013C-3513**	**SAMN08579595**	**PRJNA218110**
**E.coli**	**ERL04-3476**	**SAMN10177961**	**PRJNA494713**
**E.coli O157:H7**	**2571**	**SAMN11229478**	**PRJNA528413**
**E.coli O157:H7 str. EC4115**	**EC4115**	**SAMN02603441**	**PRJNA27739**
E.coli O157:H7	DEC5E	SAMN11229486	PRJNA528413
E.coli	AUSMDU00014361	SAMN11008224	PRJNA556438
E.coli O157:H7	F8798	SAMN05360171	PRJNA528413
E.coli O157:H7	TR01	SAMN10364854	PRJNA503568
E.coli O157:H7	ECP19-598	SAMN16946946	PRJNA681235
E.coli	ATCC 43889	SAMN01916927	PRJNA321364
**E.coli O157:H7**	**G5295**	**SAMN05360178**	**PRJNA528413**
E.coli	28RC1	SAMN04202540	PRJNA299666
E.coli	150	SAMN08773055	PRJNA445267
**E.coli O157**	**AR-0428**	**SAMN12648978**	**PRJNA562719**
E.coli O157:H7	FRIK804	SAMN10531264	PRJNA508805
E.coli	ERL05-1306	SAMN10177963	PRJNA494713
E.coli	AUSMDU00002545	SAMN13191633	PRJNA556438
E.coli O157:H7	2-6-2	SAMN05360202	PRJNA528413
E.coli O157	FDAARGOS_293	SAMN06173306	PRJNA231221
**E.coli O157:H7 str. EDL933**	**EDL933**	**SAMN02905113**	**PRJNA253471**
E.coli	ERL06-2503	SAMN10177966	PRJNA494713
E.coli O157:H7	SS TX 754-1	SAMN05360237	PRJNA528413
E.coli O157:H7	YB14-1	SAMN05360245	PRJNA528413
E.coli O157:H7	F7508	SAMN05360163	PRJNA528413
E.coli	2010C-3347	SAMN04549845	PRJNA218110
E.coli O157:H7	17B6-2	SAMN05360191	PRJNA528413
E.coli	HB6	SAMN11633027	PRJNA530317

E.coli	ECCNB12-2	SAMN07723175	PRJNA412707
E.coli O45:H2	FWSEC0003	SAMN08768104	PRJNA287560
E.coli O157	AR-0427	SAMN12648977	PRJNA562719
E.coli O157:H7	F6321	SAMN11229490	PRJNA528413
E.coli	08-3914	SAMN04504880	PRJNA218110
E.coli O157:H7 str. TW14359	TW14359	SAMN02604255	PRJNA30045
E.coli	PA20	SAMN04040761	PRJNA294158
**E.coli O157:H7**	**ECP19-2498**	**SAMN16946945**	**PRJNA681235**
E.coli O157:H7 str. SS17	SS17	SAMN02872945	PRJNA200626
E.coli O157:H7	F1273	SAMN11229487	PRJNA528413
E.coli O157:H7 str. F8092B	F8092B	SAMN02304041	PRJNA528413
E.coli O157:H7	F7349	SAMN05360158	PRJNA528413
E.coli	ERL06-2442	SAMN10177964	PRJNA494713
E.coli O157:H7	MB9-1	SAMN05360217	PRJNA528413
E.coli	9000	SAMN05544760	PRJNA336330
E.coli O157:H7	F6667	SAMN05360149	PRJNA528413
E.coli	472	SAMN05544767	PRJNA336330
E.coli O157:H7	NE 1169-1	SAMN05360232	PRJNA528413
E.coli O157:H7	NE1127	SAMN11229496	PRJNA528413
E.coli	155	SAMN05544764	PRJNA336330
E.coli O157:H7	F8492	SAMN11267279	PRJNA528413
E.coli O157:H7	LSU61	SAMN11229493	PRJNA528413
E.coli	RM19259	SAMN13429994	PRJNA573729
E.coli	2013C-4187	SAMN08579590	PRJNA218110
E.coli O157	180-PT54	SAMN05007044	PRJNA321984
E.coli	112	SAMN08773024	PRJNA445267
E.coli	111	SAMN08773023	PRJNA445267
**E.coli O157:H7**	**MB41-1**	**SAMN05360216**	**PRJNA528413**
E.coli	SRCC 1675	SAMN04202539	PRJNA299765
E.coli	144	SAMN08773051	PRJNA445267
E.coli O157:H7	N8B7-2	SAMN05360221	PRJNA528413
E.coli	ERL06-2497	SAMN10177965	PRJNA494713
**E.coli**	**NZRM4169**	**SAMN10177969**	**PRJNA494713**

Supplemental Table 2: The identifiers of the samples used in the first part of the experiment with natural E.coli samples. All data were part of bioproject PRJNA218110 and study SRP046387.

Experiment	Sample	Run
SRX6770193	SAMN12657222	SRR10035010
SRX7011058	SAMN13047823	SRR10298294
SRX7435075	SAMN13897143	SRR10760848
SRX8086834	SAMN14568886	SRR11515223
SRX8086386	SAMN14566463	SRR11514775
SRX9364727	SAMN16555685	SRR12899680
SRX10070724	SAMN17861353	SRR13681366
SRX10990244	SAMN19336591	SRR14651731
SRX2194368	SAMN05788720	SRR4299818
SRX2662962	SAMN06624388	SRR5367694
SRX3058572	SAMN07429387	SRR5892897
SRX3349343	SAMN07662592	SRR6241970
SRX3770854	SAMN08646861	SRR6813864
SRX4175059	SAMN09375722	SRR7271452
SRX4518650	SAMN09779958	SRR7656265
SRX4745878	SAMN10139043	SRR7909757
SRX5074318	SAMN10492716	SRR8256810
SRX5554100	SAMN11192045	SRR8763187
SRX5796702	SAMN11582321	SRR9018582
SRX6453210	SAMN12287265	SRR9694889

Supplemental Table 3: The identifiers of the samples used in the second part of the experiment with natural E.coli samples. All data were part of bioproject PRJNA218110 and study SRP046387.

Experiment	Sample	Run	Strain
SRX7116262	SAMN13242703	SRR10418234	2016AM-1919
SRX7116264	SAMN13242707	SRR10418236	2016AM-0680
SRX7116266	SAMN13242725	SRR10418238	2016AM-0781
SRX7116271	SAMN13242726	SRR10418243	2016AM-0587
SRX7116302	SAMN13242766	SRR10418274	2016AM-0812
